# Effectiveness of varicella vaccine during a varicella outbreak in a Chinese senior high school: a retrospective cohort study

**DOI:** 10.3389/fimmu.2026.1847492

**Published:** 2026-07-21

**Authors:** Nani Xu, Liting Wu, Xuan Deng, Xin Shu, Yejing Yang, Pan Qin, Kailuo Zhong, Shenyu Wang, Xiaowei Hu

**Affiliations:** 1 Department of Immunization Program, Xihu District Center for Disease Control and Prevention(Xihu District Health Inspection), Hangzhou, China; 2 Zhejiang Provincial Center for Disease Control and Prevention, Hangzhou, China; 3 Zhejiang Academy of Preventive Medicine, Hangzhou, China; 4 Department of Social Medicine of School of Public Health, and Department of Pharmacy of the First Affiliated Hospital, Zhejiang University School of Medicine, Hangzhou, Zhejiang, China

**Keywords:** immunization strategy, outbreak, vaccine effectiveness, varicella, varicella vaccine

## Abstract

**Objective:**

To evaluate the vaccine effectiveness (VE) of single-dose and two-dose varicella vaccine (VarV) among senior high school students during a varicella outbreak in China, and provide recommendations for vaccination strategy optimization.

**Methods:**

A retrospective cohort study was conducted among students in a senior high school in China during a varicella outbreak from July 23 to November 8, 2024. The history of varicella disease, the immunization history of VarV and the incidence during the outbreak among all students in the school were surveyed. Kaplan–Meier methods were used to estimate the cumulative varicella-free survival rate among students with different immunization histories during the outbreak, and the log-rank test was used to compare the survival curves across the five groups. VE was calculated based on attack rates among students with different vaccination doses and intervals since last vaccination. Post-exposure vaccination VE was also assessed. Logistic regression analysis was performed to identify factors associated with varicella infection during the outbreak, and odds ratio (OR) and 95% confidence interval (95% *CI*) were calculated.

**Results:**

Among the 1,862 students present during the outbreak, the overall varicella attack rate was 6.29%. Kaplan–Meier analysis showed significant differences in cumulative varicella-free survival among the different immunization groups (*χ*² = 297.913, *df* = 4, *P* < 0.001). Single-dose varicella vaccine (VarV) coverage was 28.24% (475/1682), and two-dose coverage was 66.23% (1114/1682). Compared with unvaccinated individuals, VE with 95% *CI* was 32.14% (−54.37%- 70.17%) for 1-dose recipients and 94.74% (88.62%-97.57%) for 2-dose recipients. Among 1-dose recipients, VE was 90.48% (17.22%-98.90%) and 18.68% (−86.86%- 64.61%) for those with an immunization interval ≤10 years and >10 years, respectively. Among 2-dose recipients, VE was 95.94%(90.69%-98.23%) and 92.89%(83.87%-96.87%) for those with an immunization interval ≤6 years and >6 years, respectively. Logistic regression showed that male students had a higher risk of varicella than female students; prior receipt of two doses of VarV significantly reduced the risk of infection; and the risk increased by 1 year for each additional year between the last immunization and exposure to the index case. The VE of post-exposure vaccination was 90.15% (68.53%-96.92%) among unvaccinated students and 94.88% (89.47%-97.51%) among students who had previously received 1 dose.

**Conclusion:**

A single dose of varicella vaccine provides limited long-term protection among high school students, whereas a two-dose regimen offers robust and sustained effectiveness. Routine catch-up vaccination and timely post-exposure vaccination are recommended for the effective control of varicella outbreaks in school settings.

## 1 Introduction

Varicella is an acute contagious respiratory disease caused by primary infection with the varicella-zoster virus (VZV) (1). The main clinical features of varicella are generalized pruritic vesicles, and it may lead to a series of complications, including skin bacterial superinfection, encephalitis and pneumonitis, which can result in hospitalization or even death in severe cases (2). Owing to its high contagiousness, varicella can easily cause clustered outbreaks in kindergartens, schools and other child gathering settings (3), imposing a substantial burden on families and public health systems. Vaccination with varicella vaccine (VarV) is the most effective measure for the prevention of varicella (4, 5). Since 1998, Zhejiang Province has introduced VarV as a non-expanded program on immunization(non-EPI) vaccine and implemented a single-dose vaccination schedule. With the prolongation of time after immunization, the vaccine effectiveness(VE) of the single-dose vaccination declines (6), and Breakthrough varicella(BV) persisted in schools and kindergartens with high single-dose vaccination coverage (7, 8). Accordingly, Zhejiang Province, China recommended a two-dose vaccination schedule for children, with the first dose administered at 15 months of age and the second at 3 years of age in 2014. However, a 2020 survey showed that only 11.43% of children aged 1–14 years had completed the two-dose vaccination regimen in China (9), which is far below the 80% vaccination coverage recommended by the World Health Organization (10).

Varicella predominantly occurs in children under 15 years of age, but studies have identified a current trend toward an older age distribution (11–13). This shift means that a growing number of cases are now being observed in adolescents. According to Lyu (14), across distinct educational stages, senior high school students, despite having developed a certain level of basic immunity compared to young children with immature immune systems, still face high varicella transmission in classrooms. Concurrently, high school environments, characterized by high population density and prolonged close contact (15), create favorable conditions for disease transmission, even among vaccinated individuals (16). A study also noted that the age shift became prominent from 2020 onward: the 10–19 year-old group gradually becoming the primary population affected by varicella (17). Nevertheless, empirical data on VarV effectiveness among Chinese high school students during actual outbreak scenarios remain limited.

In this study, we performed a retrospective cohort study to evaluate and compare the VE of single-dose and two-dose VarV among high school students during a varicella outbreak at a senior high school in Zhejiang Province, China, in order to provide evidence for optimizing varicella vaccination strategies.

## 2 Materials and methods

### 2.1 Study design and participants

All students enrolled in a senior high school in Zhejiang Province,China, between July 23 and November 8, 2024, a period covering the entire course from the onset to the termination of a varicella outbreak, were recruited as participants in this epidemiological investigation.

In this study, varicella cases were identified via the China Information System for Disease Control and Prevention and symptom-based surveillance (morning and midday checks) during the outbreak period. Epidemiological investigations were conducted on all identified cases, involving the collection of basic demographic data, clinical manifestations, prior history of varicella infection, and VarV immunization status. Concurrently, a retrospective survey was conducted among all students in the school to confirm their prior history of varicella infection and VarV immunization status. Specifically, VarV immunization status was retrieved from the “Zhejiang Provincial Intelligent Vaccination Service Information System” and cross-validated with students’ paper-based vaccination certificates.

### 2.2 Define

According to *Diagnosis and Treatment Plan for Varicella (2023 Edition) (*18) published by the National Health Commission of the People’s Republic of China, clinically diagnosed cases were defined as those meeting any one of the following criteria: 1.Presence of typical clinical manifestations (acute onset of diffuse or generalized macules, papules, vesicles, and crusts appearing in crops, with superficial location, thin and fragile vesicle walls, and surrounding erythematous halos), 2.Presence of atypical clinical manifestations but with a clear epidemiological history (contact with varicella or herpes zoster patients within 3 weeks before onset). Diagnosis was established based on a comprehensive analysis of epidemiological history, clinical manifestations, laboratory tests, and other relevant findings.

Breakthrough varicella (BV) is defined as an infection occurring more than 42 days after vaccination (7).

Breakthrough interval: The time interval between first varicella vaccination and the occurrence of varicella infection. Calculation formula: Breakthrough interval (years) = Date of varicella onset - Date of last varicella vaccination.

Immunization interval: The time interval between the date of onset of the index case in the class and the date of last VarV dose. For students in classes with no cases, the interval was calculated based on the onset date of the index case in the nearest class with cases.

### 2.3 Statistical analysis

Data were collated using WPS Excel 2024, SPSS 25.0 and R4.2.3 software performed statistical analyses. Descriptive statistical analyses, including frequency and constituent ratio, were performed on the case characteristics. Kaplan–Meier methods were used to estimate the cumulative varicella-free survival rate among students with different immunization histories during the outbreak, and the log-rank test was used to compare the Kaplan–Meier curves among the 0-dose, 1-dose, 2-dose, 0 + 1-dose, and 1 + 1-dose groups. The chi-square test or Fisher’s exact test was used to compare the differences in varicella attack rates between groups. VE was calculated using the following formula: [1 - relative risk (RR)]×100%. Logistic regression analysis was used to identify factors associated with varicella infection during this outbreak, and odds ratios (ORs) and their 95% *CI* were calculated. Statistical significance was set at *P* < 0.05.

## 3 Results

### 3.1 Characteristics of the school

This senior high school is a public semi-boarding institution, consisting of three grades with a total of 40 classes and 1,910 enrolled students. However, due to student absences from campus attributed to reasons such as off-campus training, sick leave (for non-varicella-related illnesses), or overseas study, a total of 1,861 students were present on campus during the outbreak period, among whom 792 were boarding students. The grade-specific distribution of present students was as follows: Grade 1 had 13 classes with 632 students present, Grade 2 had 14 classes with 662 students present, and Grade 3 had 13 classes with 567 students present. All students consumed meals provided by the school cafeteria, which were served at staggered times by grade level. Student mixing between different classes occurred during cross-class courses.

### 3.2 Characteristics of varicella cases

**Figure 1:**
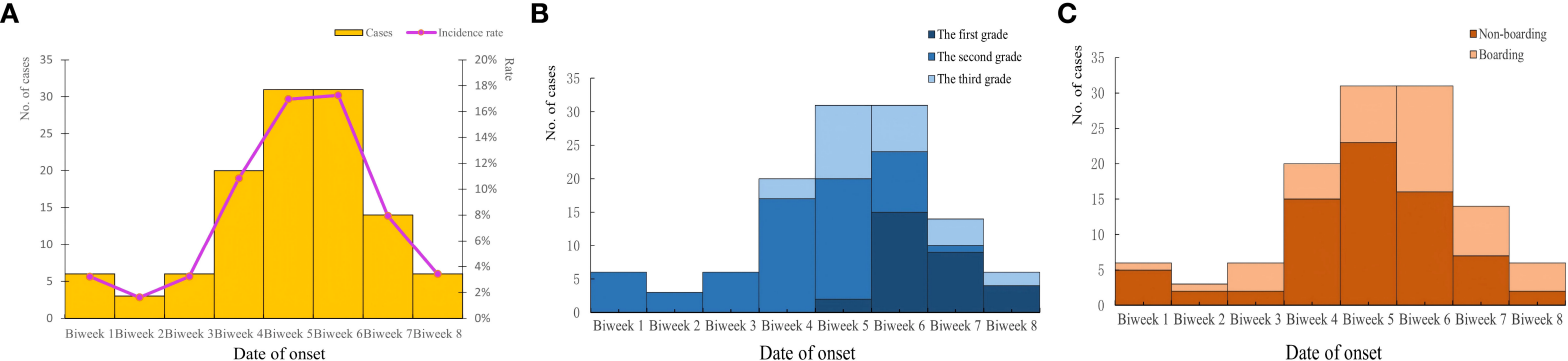
Temporal distribution of cases by grade and boarding status *(A) Showing incidence rate, **(B)** Distribution by grade, **(C)** Distribution by boarding status Biweek 1 (07/23-08/05), Biweek 2 (08/06-08/19), Biweek 3 (08/20-09/02), Biweek 4 (09/03-09/16), Biweek 5 (09/17-09/30), Biweek 6 (10/01-10/14), Biweek 7 (10/15-10/28), Biweek 8 (10/29-11/09).

A total of 117 varicella cases were reported in this outbreak ranging from July 23 to November 8, 2024, yielding an overall attack rate of 6.29%. Among the reported cases, 75 were male and 42 were female, with an average age of 16.6 years. By grade level, 30 cases (25.64%) occurred in 7 classes of Grade 1, 60 cases (51.28%) in 13 classes of Grade 2, and 27 cases (23.08%) in 8 classes of Grade 3. With respect to residential status, 72 cases (61.54%) were identified among non-boarding students, as shown in **Figure 1**. All cases were outpatient, except for two commuting students who required hospitalization. A series of control measures were implemented during this outbreak, including enhanced health monitoring, case isolation, emergency VarV vaccination, disinfection and ventilation, suspension of mass gatherings, and health education. The outbreak lasted 128 days from onset to resolution. No additional cases were detected during the 21-day observation period (the maximum incubation period) following onset of the last two cases, and the outbreak was declared over.

### 3.3 VarV vaccination status among students on campus

**Figure 2:**
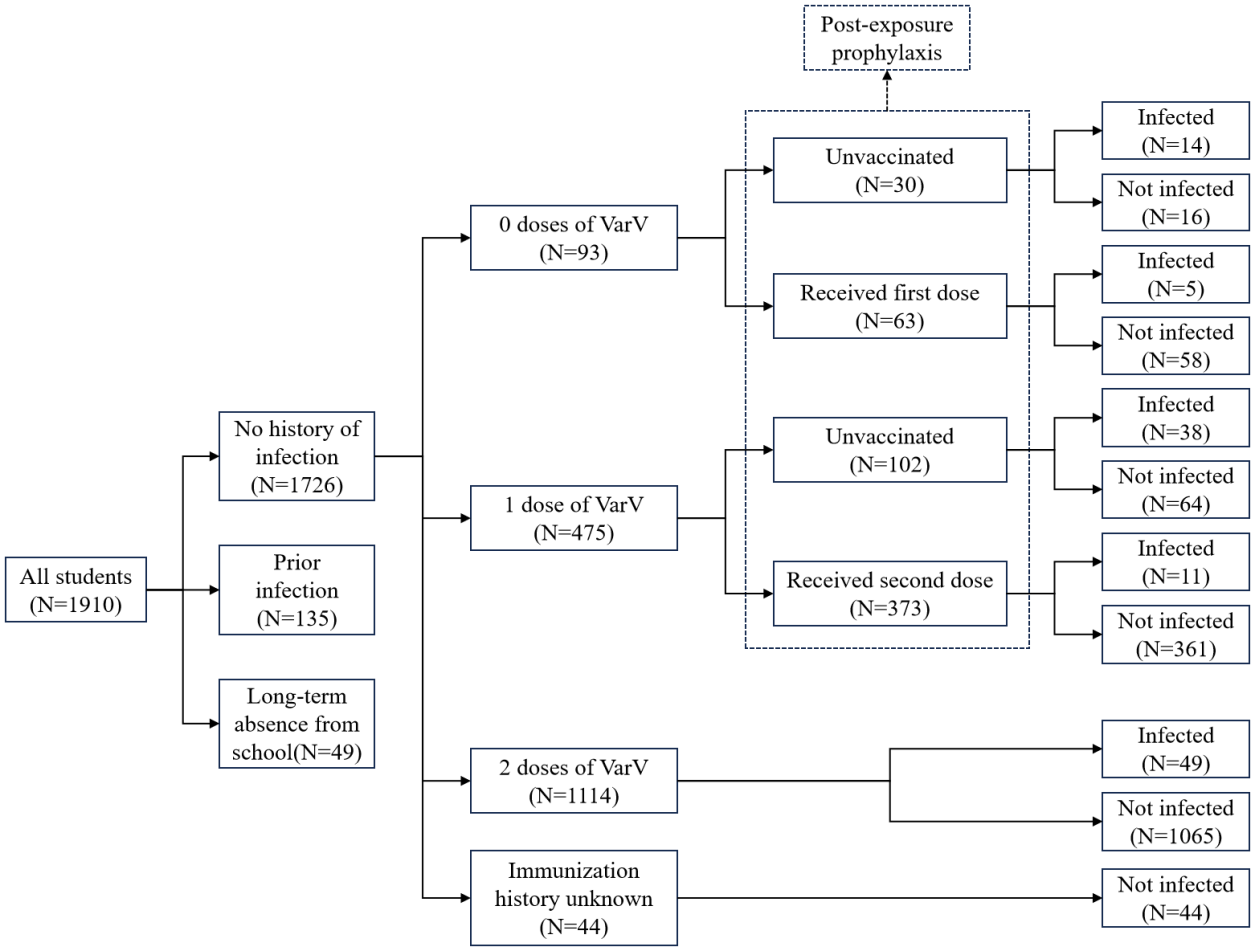
VarV vaccination status and varicella incidence among students during the outbreak.

Based on epidemiological investigation findings, among the 1,862 students present on campus during the outbreak, 135 had a previous history of varicella infection. Of the 1,726 students with no prior varicella infection, 44 had unknown immunization status (with no relevant records in the Zhejiang Provincial Intelligent Vaccination Service Information System and no paper vaccination certificates available), and 93 students had received zero doses of VarV. Among students with documented immunization histories, the coverage rate of VarV was 94.47% (1589/1682). Specifically, the single-dose VarV vaccination rate was 28.24% (475/1682), while the two-dose vaccination rate reached 66.23% (1114/1682). During the outbreak, 436 students received VarV as post-exposure prophylaxis, including 63 individuals administered their first dose and 373 their second dose, as shown in **Figure 2**.

Of the 117 varicella cases, 19 (16.24%) had received 0 doses of VarV previously, including 5 individuals who developed the disease following their first dose administered as post-exposure prophylaxis. The remaining 98 cases (83.76%) were BV, of whom 49 had a history of single-dose VarV vaccination (including 11 who developed the disease after a dose given as post-exposure prophylaxis) and 49 had a history of two-dose VarV vaccination,as shown in **Figure 2**. Additionally, a total of 16 individuals developed varicella after receiving VarV vaccination during the outbreak, the median interval between vaccination and breakthrough infection was 10 days, ranging from a minimum of 6 days to a maximum of 15 days.

### 3.4 Survival analysis of varicella infection among students with different vaccination histories

**Figure 3:**
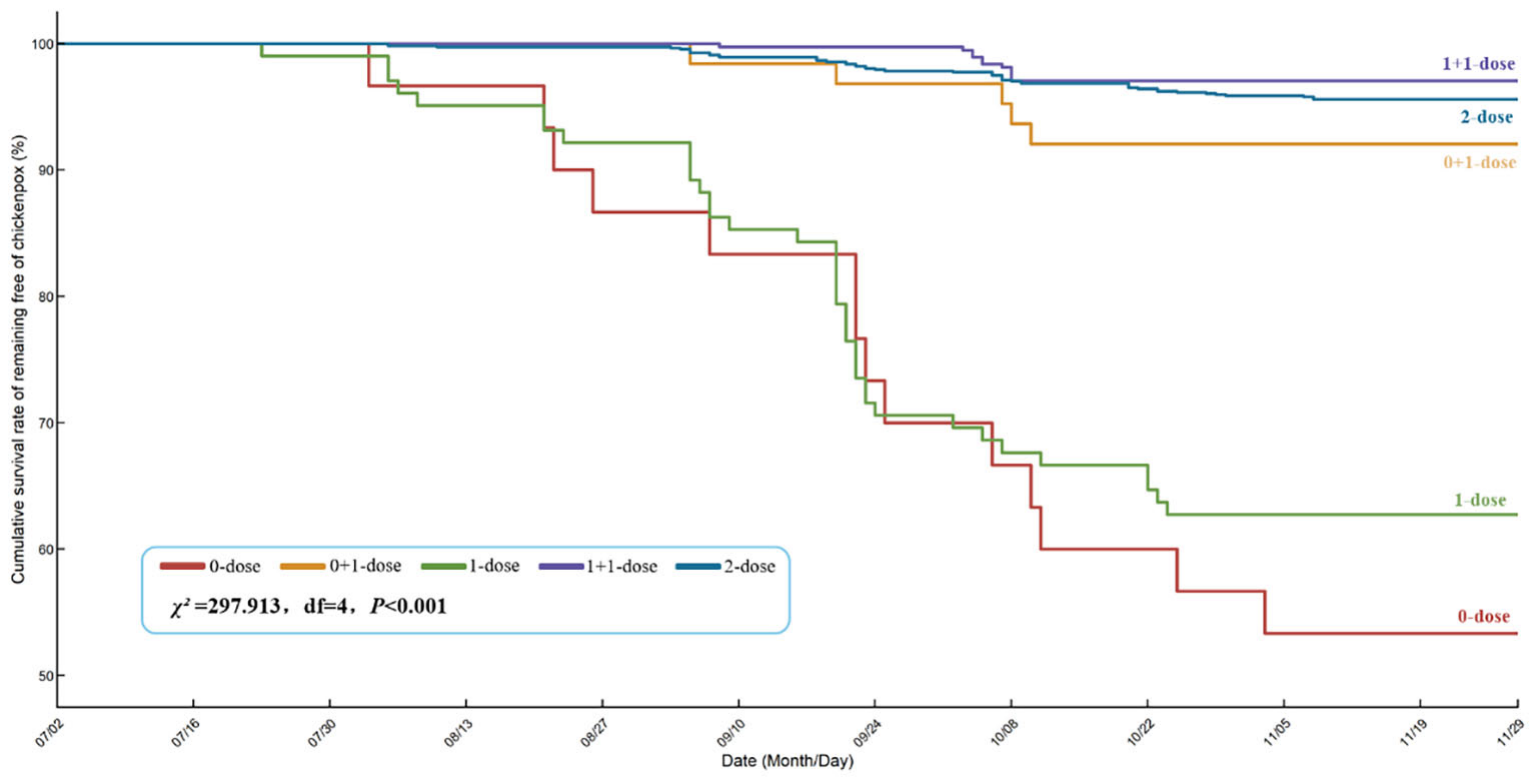
Survival curves for varicella among students with different immunization histories. The 0 + 1-dose group refers to students who had not received varicella vaccine before the outbreak and received one dose during the outbreak; the 1 + 1-dose group refers to students who had received one dose before the outbreak and received one additional dose during the outbreak.

The cumulative varicella-free survival curves differed significantly among the different vaccination groups, and the between-group comparison was statistically significant (*χ²* = 297.913, *df* = 4, *P* < 0.001). Overall, the unvaccinated group showed the steepest decline in cumulative varicella-free survival, indicating the highest cumulative risk of varicella during follow-up. The one-dose group had a higher cumulative varicella-free survival rate than the unvaccinated group, but it remained lower than that of the other vaccination groups. In contrast, the 0 + 1-dose, 1 + 1-dose, and 2-dose groups maintained relatively high cumulative varicella-free survival rates throughout follow-up, suggesting that emergency vaccination and higher-dose vaccination were associated with a lower risk of varicella infection. In particular, the 1 + 1-dose and 2-dose groups consistently showed high survival rates over time, indicating that additional doses administered during the outbreak may have provided better protection. As shown in **Figure 3**.

### 3.5 VE with different immune histories

Among students with no prior VarV immunization (excluding those who received post-exposure prophylaxis; hereinafter the same), the varicella attack rate was 46.67%. Among students with single-dose VarV immunization, the immunization interval was 15.20 (95% *CI:* 14.19-15.77) years, with an attack rate of 37.25% and a corresponding VE of 32.14% (95% *CI:* -54.37%-70.17%) (*χ*²=0.860, *P* = 0.354). Among students with two-dose VarV immunization, the immunization interval was 5.21 (95% *CI:* 2.27-9.48) years, with an attack rate of 4.40% and a corresponding VE of 94.74% (95% *CI:* 88.62%-97.57%) *(χ*²=100.298, *P* < 0.001). As shown in **Table 1**. Moreover, the difference in vaccine effectiveness between the two-dose and single-dose regimens was statistically significant (*χ*²=151.86, *P* < 0.001).

**Table 1:** Analysis of VE with different vaccination histories and immunization intervals.

Immune history	Immunization interval (years)	Exposed cases	Infected cases	Attack rate (%)	Relative risk (95% CI)	VE (%) (95% CI)	*χ*²	*P*
0-dose	–	30	14	46.67	–	–	–	–
1-dose (vs. 0-dose group)	1-dose	102	38	37.25	0.679 (0.298, 1.544)	32.14 (-54.37, 70.17)	0.860	0.354
	≤10	13	1	7.69	0.095 (0.011, 0.828)	90.48 (17.22, 98.90)	–	0.017^*^
	>10	89	37	41.57	0.813 (0.345, 1.869)	18.68 (-86.86, 64.61)	0.238	0.626
2-dose (vs. 0-dose group)	2-dose	1114	49	4.40	0.053 (0.024, 0.114)	94.74 (88.62, 97.57)	–	<0.001^*^
	≤6	670	23	3.43	0.041 (0.018, 0.093)	95.94 (90.69, 98.23)	–	<0.001^*^
	>6	444	26	5.86	0.071 (0.031, 0.161)	92.89 (83.87, 96.87)	–	<0.001^*^

* Using Fisher’s exact test.

Among single-dose recipients, the VE was significantly associated with immunization interval. Attack rates were 37.25% and 7.69% for immunization intervals ≤10 years and >10 years, respectively (*P* = 0.028), with corresponding VE of 90.48%(95% CI: 17.22%, 98.90%) *(P* = 0.017) and 18.68% (95% *CI:* -86.86%-64.61%) *(χ*²=0.238, *P* = 0.626). Among two-dose recipients, attack rates were 3.43% and 5.86% for immunization intervals ≤6 years and >6 years, respectively(*P* = 0.053), with corresponding VE of 95.94%(95% *CI:* 90.69%-98.23%) *(P* < 0.001) and 92.89%(95% *CI:* 83.87%-96.87%) *(P* < 0.001).

### 3.6 VE of post-exposure prophylaxis

During this outbreak, 63 students with no prior VarV receipt received post-exposure VarV vaccination, 5 cases were identified (all within 14 days post-vaccination), with an attack rate of 7.94% and VE of 90.15% (95% *CI:* 68.53%-96.92%)(*χ*²=18.752, *P* < 0.001). Among students with 1 prior VarV dose, 373 received post-exposure vaccination, with 11 cases reported (10 within 14 days and 1 on day 15 post-vaccination), yielding an attack rate of 2.95% and a VE of 94.88% (95% *CI:* 89.47%-97.51%)(*χ*²=101.890, *P* < 0.001). As shown in **Table 2**.

**Table 2:** Analysis of vaccine effectiveness after exposure.

Vaccination status	Exposed cases	Infected cases	Attack rate (%)	Relative risk (CI)	VE (%) (95% *CI*)	*χ*²	*P*
0-dose	30	14	46.67	–	–	–	–
0 + 1-dose	63	5	7.94	0.099 (0.031- 0.315)	90.15 (68.53-96.62)	18.752	<0.001
1-dose	102	38	37.25	–	–	–	–
1 + 1-dose	373	11	2.95	0.051 (0.025-0.105)	94.88 (89.47-97.51)	101.890	<0.001

Group definitions are the same as in **Figure 3**.

### 3.7 Logistic regression analysis of factors associated with varicella infection among students with routine vaccination

In the univariate logistic regression analysis, male students had a significantly higher risk of varicella than female students (*OR* = 2.13, 95% *CI*: 1.39-3.26, *P* < 0.001). Regarding vaccination history, compared with unvaccinated students, those who had received one dose showed no significant difference in risk (*OR* = 0.68, 95% *CI*: 0.30-1.54, *P* = 0.355), whereas those who had received two doses had a significantly lower risk (*OR* = 0.05, 95% *CI*: 0.02-0.11, *P* < 0.001). In addition, each additional prior vaccine dose was associated with a further reduction in risk (*OR* = 0.12, 95% *CI*: 0.02-0.66, *P* = 0.015). The risk of varicella increased with each additional year between the last vaccination and exposure to the index case (*OR* = 1.33, 95% *CI*: 1.25-1.41, *P* < 0.001). As shown in **Table 3**.

After adjustment for sex, age, vaccination history, and the interval between the last vaccination and exposure to the index case, multivariable logistic regression showed that male sex remained an independent risk factor for varicella (*OR* = 2.49, 95% *CI*: 1.51-4.11, *P* < 0.001). Age was not significantly associated with varicella risk (*OR* = 0.97, 95% *CI*: 0.72-1.31, *P* = 0.837). Compared with unvaccinated students, those who had received one dose still showed no significant difference in risk (*OR* = 0.68, 95% *CI*: 0.30-1.58, *P* = 0.374), whereas those who had received two doses had a significantly lower risk, both compared with the unvaccinated group (*OR* = 0.05, 95% *CI*: 0.02-0.12, *P* < 0.001) and compared with the one-dose group (*OR* = 0.27, 95% *CI*: 0.12-0.59, *P* < 0.001). The risk of varicella increased by 19% for each additional year between the last vaccination and exposure to the index case (*OR* = 1.19, 95% *CI*: 1.09-1.29, *P* < 0.001), suggesting that vaccine protection may wane over time. As shown in **Table 3**.

**Table 3:** Logistic regression analysis of factors associated with varicella infection among students with routine vaccination.

Analysis type	Variable	Comparison	Adjusted variables	*OR* (95% *CI*)	*P* value
Univariate analysis	Sex	Male vs Female	–	2.13(1.39-3.26)	<0.001
Univariate analysis	Age	Per 1-year increase	–	1.08(0.85-1.38)	0.515
Univariate analysis	Previous immunity history	1-dose vs 0-dose	–	0.68(0.30-1.54)	0.355
Univariate analysis	Previous immunity history	2-dose vs 0-dose	–	0.05(0.02-0.11)	<0.001
Univariate analysis	Previous immunity history	Per 1-dose increas	–	0.12(0.02-0.66)	0.015
Univariate analysis	Interval between last vaccination and exposure to the index case	Per 1-year increase	–	1.33(1.25-1.41)	<0.001
Multivariate analysis	Sex	Male vs Female	Age, previous immunity history, interval between last vaccination and exposure to the index case	2.49(1.51-4.11)	<0.001
Multivariate analysis	Age	Per 1-year increase	Sex, previous immunity history, interval between last vaccination and exposure to the index case	0.97(0.72-1.31)	0.837
Multivariate analysis	Previous immunity history	1-dose vs 0-dose	Sex, age	0.68(0.30-1.58)	0.374
Multivariate analysis	Previous immunity history	2-dose vs 0-dose	Sex, age	0.05(0.02-0.12)	<0.001
Multivariate analysis	Previous immunity history	2-dose vs 1-dose	Sex, age, interval between last vaccination and first exposure	0.27(0.12-0.59)	<0.001
Multivariate analysis	Interval between last vaccination and exposure to the index case	Per 1-year increase	Sex, age, previous immunity history	1.19(1.09-1.29)	<0.001

Group definitions are the same as in **Figure 3**.

### 3.8 Logistic regression analysis of factors associated with varicella infection among students with emergency vaccination

Among students who received emergency vaccination, univariate logistic regression showed that compared with unvaccinated students, those who received 0 + 1 dose had a significantly lower risk of varicella (*OR* = 0.10, 95% *CI*: 0.03-0.31, *P* < 0.001). Compared with students who received 0 + 1 dose, those who received 1 dose had a significantly higher risk (*OR* = 6.89, 95% *CI*: 2.54-18.68, *P* < 0.001). In addition, compared with students who received 1 dose, those who received 1 + 1 doses had a significantly lower risk (*OR* = 0.05, 95% *CI*: 0.02-0.11, *P* < 0.001). However, no statistically significant difference was observed between students who received 2 doses and those who received 1 + 1 doses (*OR* = 1.51, 95% *CI*: 0.78-2.94, *P* = 0.221). As shown in **Table 4**.

After adjustment for sex and age, the multivariable logistic regression results remained consistent with the univariate findings. Compared with unvaccinated students, those who received 0 + 1 dose still had a significantly lower risk of varicella (*OR* = 0.09, 95% *CI*: 0.03-0.30, *P* < 0.001). Compared with students who received 0 + 1 dose, those who received 1 dose had a significantly higher risk (*OR* = 6.89, 95% *CI*: 2.54-18.72, *P* < 0.001). Compared with students who received 1 dose, those who received 1 + 1 doses had a significantly lower risk (*OR* = 0.05, 95% *CI*: 0.02-0.11, *P* < 0.001). In contrast, no significant difference was found between students who received 2 doses and those who received 1 + 1 doses (*OR* = 1.51, 95% *CI*: 0.77–2.96, *P* = 0.230).

Overall, emergency vaccination was associated with a reduced risk of varicella among students, although the protective effect varied across vaccination patterns. As shown in **Table 4**.

**Table 4:** Logistic regression analysis of factors associated with varicella infection among students with emergency vaccination.

Analysis type	Comparison	Adjusted variables	*OR* (95% *CI*)	*P* value
Univariate analysis	0 + 1-dose vs 0-dose	–	0.10(0.03-0.31)	<0.001
Univariate analysis	1-dose vs 0 + 1-dose	–	6.89(2.54-18.68)	<0.001
Univariate analysis	1 + 1-dose vs 1-dose	–	0.05(0.02-0.11)	<0.001
Univariate analysis	2-dose vs 1 + 1-dose	–	1.51(0.78-2.94)	0.221
Multivariate analysis	0 + 1-dose vs 0-dose	Sex, Age	0.09(0.03-0.30)	<0.001
Multivariate analysis	1-dose vs 0 + 1-dose	Sex, Age	6.89(2.54-18.72)	<0.001
Multivariate analysis	1 + 1-dose vs 1-dose	Sex, Age	0.05(0.02-0.11)	<0.001
Multivariate analysis	2-dose vs 1 + 1-dose	Sex, Age	1.51(0.77-2.96)	0.230

Group definitions are the same as in **Figure 3**.

## 4 Discussion

VarV vaccination coverage among students at the involved high school during this outbreak was 94.47% (1589/1682). Of these, 1114 students received a second dose following Zhejiang Province’s implementation of the two-dose VarV schedule in 2014, corresponding to a two-dose coverage rate of 66.23% (1114/1682), which was higher than rates reported in previous studies (9, 19–21).

The results of this study showed that VE among recipients of a single prior VarV dose was 32.14%. VE was 90.48%(95% *CI*: 17.22%-98.90%) (P = 0.017) and 18.68% (95% *CI:* -86.86%, 64.61%) (*χ*²=0.238, *P* = 0.626) among those with an immunization interval of ≤10 years and >10 years respectively. These findings indicate that antibody levels decline substantially by 10 years after a single VarV dose, resulting in inadequate long-term protective immunity. We conducted univariate and multivariate analyses regarding the waning of vaccine-induced protection. The results showed that the risk of varicella rose by 19% for each additional year from the last vaccination to exposure to the index case (OR = 1.19, 95% *CI:* 1.09-1.29, *P* < 0.001), indicating that vaccine protection tends to wane over time. Waning immunity is considered a contributing factor to this outbreak. A Systematic Review and Meta-Analysis and two real-world studies on varicella conducted in Guangzhou, China have also confirmed this observation (22–24).

In our research, multivariable logistic regression showed that male students had a higher risk of varicella than female students(OR = 2.49, 95% *CI:* 1.51-4.11, P < 0.001). These findings are consistent with previous varicella epidemiological studies conducted by the Chinese Center for Disease Control and Prevention (China CDC) (25). Similar findings have also been reported in a cohort study of school-aged children in Shanghai (26). Higher frequency of close group activities among male adolescents may explain the elevated infection risk in male students (25, 26).

Compared with unvaccinated students, those with two-dose VarV immunization had a lower attack rate. VE was 95.94%(95% *CI*: 90.69%-98.23%) (P<0.001) and 92.89%(95% *CI:* 83.87%-96.87%) (P<0.001) for immunization intervals of ≤6 years and >6 years between last vaccination and exposure, respectively. These results suggest that the two-dose VarV schedule maintains high and sustained VE, with no obvious decline over the observed interval (27, 28).

VarV vaccination is an effective measure for the prevention and control of varicella outbreaks (29, 30). Following this outbreak, 436 students received post-exposure VarV vaccination, among whom 16 breakthrough cases were identified. The median interval between vaccination and breakthrough infection was 10 days, including 15 cases with symptom onset within 14 days and 1 case developing symptoms on day 15 post-vaccination. Therefore, we also included cases with symptom onset within 14 days in the analysis. As VarV generally induces protective immunity approximately two weeks after vaccination (31, 32), This inevitably leads to an underestimation of the protective effect of emergency varicella vaccination. Despite this, our study demonstrated that post-exposure VE reached 90.15%(95% *CI:* 68.53%-96.92%)(*χ*²=18.752, *P* < 0.001) and 94.88%(95% *CI:* 89.47%-97.51%)(*χ*²=101.890, *P* < 0.001) among students with 0-dose and 1-dose immunization histories, respectively. These findings indicate that post-exposure VarV vaccination represents an effective intervention for controlling varicella outbreaks.

The main limitations of this study are as follows. First, 40 students with unknown vaccination records were excluded from the analysis, which may have introduced selection bias. Second, we did not further investigate or analyze the clinical symptoms and severity of varicella cases, thus preventing an assessment of whether VarV vaccination mitigates disease severity. In addition, the diagnosis of varicella cases in medical institutions currently relies predominantly on clinical diagnosis, with laboratory confirmation rarely performed in China. Therefore, misclassification bias may indeed be introduced into the results of this study, which constitutes one of the limitations of the present paper.

## 5 Conclusion

A single dose of VarV offers limited long-term protection among high school students, whereas a two-dose VarV vaccination strategy effectively reduces the incidence of varicella. Routine VarV catch-up vaccination is recommended for this population. In the event of a varicella outbreak, post-exposure vaccination should be implemented promptly to effectively control transmission and minimize adverse impacts on students’ health and academic activities.

## Data Availability

The dataset used in this study is restricted to the research purposes described in the article only. Due to privacy and ethical restrictions (e.g., protecting the personal information of senior high school students, including their vaccination and disease history), the dataset is not publicly available. Requests for data access can be directed to the corresponding author with a reasonable research proposal and ethical approval.

## References

[1] Zhou H Long Q Xie H Yao Y Deng C . Epidemiological characteristics of breakthrough varicella cases among students in Jiulongpo District, Chongqing: implications for vaccination strategies. Front Immunol. (2025) 16:1594598. doi: 10.3389/fimmu.2025.1594598 40630958 PMC12235188

[2] Kennedy PGE Gershon AA . Clinical features of varicella-zoster virus infection. Viruses. (2018) 10:609. doi: 10.3390/v10110609 30400213 PMC6266119

[3] Zhu H Zhao H Ou R Zeng Q Hu L Qiu H . Spatiotemporal epidemiology of varicella in Chongqing, China, 2014-2018. Int J Environ Res Public Health. (2020) 17:662. doi: 10.3390/ijerph17020662 31968545 PMC7013978

[4] Zhang L Ma W Liu Y Wang Y Sun X Hu Y . Analysis of sero-epidemiological characteristics of varicella in healthy children in Jiangsu Province, China. BMC Infect Dis. (2018) 18:563. doi: 10.1186/s12879-018-3496-8 30428851 PMC6234534

[5] Smith-Norowitz TA Saadia TA Norowitz KB Joks R Durkin HG Kohlhoff S . Negative IgG varicella zoster virus antibody status: immune responses pre and post re-immunization. Infect Dis Ther. (2018) 7:175–81. doi: 10.1007/s40121-017-0182-x 29273977 PMC5840101

[6] Zhang Z Suo L Pan J Zhao D Lu L . Two-dose varicella vaccine effectiveness in China: a meta-analysis and evidence quality assessment. BMC Infect Dis. (2021) 21:543. doi: 10.1186/s12879-021-06217-1 34107891 PMC8188742

[7] Chartrand SA . Varicella vaccine. Pediatr Clin North Am. (2000) 47:373–94. doi: 10.1016/s0031-3955(05)70212-1 10761509

[8] Zhang X Yan Y Liu F Luo Y Zhang J Peng X . The epidemiology and risk factors for breakthrough varicella in Beijing Fengtai district. Vaccine. (2012) 30:6186–9. doi: 10.1016/j.vaccine.2012.07.062 22867721

[9] Hu Q Zhang Q Li Y Zheng H Liu Q Tang L . Varicella vaccine coverage levels among 1-14-year-old children in China in 2020: a cross-sectional survey. Chin J Vaccines Immunization. (2022) 28(2):169–173178. doi: 10.19914/j.CJVI.2022033

[10] Wutzler P Bonanni P Burgess M Gershon A Sáfadi MA Casabona G . Varicella vaccination - the global experience. Expert Rev Vaccines. (2017) 16:833–43. doi: 10.1080/14760584.2017.1343669 28644696 PMC5739310

[11] Gershon AA Breuer J Cohen JI Cohrs RJ Gershon MD Gilden D . Varicella zoster virus infection. Nat Rev Dis Primers. (2015) 1:15016. doi: 10.1038/nrdp.2015.16 27188665 PMC5381807

[12] Xu Y Liu Y Zhang X Zhang X Du J Cai Y . Epidemiology of varicella and effectiveness of varicella vaccine in Hangzhou, China, 2019. Hum Vaccin Immunother. (2021) 17:211–6. doi: 10.1080/21645515.2020.1769395 32574100 PMC7872021

[13] Suzuki A Nishiura H . Reconstructing the transmission dynamics of varicella in Japan: an elevation of age at infection. PeerJ. (2022) 10:e12767. doi: 10.7717/peerj.12767 35111401 PMC8783564

[14] Lyu H Chen M Zhou H Zhang Z Zeng Y Xiong H . Analysis and comparison of chickenpox outbreaks in two schools at different educational stages: a mixed retrospective observational study. BMC Infect Dis. (2025) 25:1543. doi: 10.1186/s12879-025-11981-5 41219850 PMC12606901

[15] Wang M Li X You M Wang Y Liu X Li Z . Epidemiological characteristics of varicella outbreaks - China, 2006-2022. China CDC Wkly. (2023) 5:1161–6. doi: 10.46234/ccdcw2023.218 38164468 PMC10757729

[16] Tugwell BD Lee LE Gillette H Lorber EM Hedberg K Cieslak PR . Chickenpox outbreak in a highly vaccinated school population. Pediatrics. (2004) 113:455–9. doi: 10.1542/peds.113.3.455 14993534

[17] Gu L Liu Y Zhang X Xu Y Zhang X Che X . Spatial-temporal patterns, seasonality, and age-specific trends of varicella in Hangzhou, China, 2019-2024. Front Public Health. (2026) 14:1701894. doi: 10.3389/fpubh.2026.1701894 41694520 PMC12901367

[18] National Health Commission of the People’s Republic of China State Administration of Traditional Chinese Medicine . Diagnosis and treatment plan for varicella (2023 edition). Int J Epidemiol Infect Dis. (2024) 51(1):4–6. doi: 10.3760/cma.j.cn331340-20240117-00013 30704229

[19] Fang Q Yang H Wang S Gong X Zheng C Yin Z . Impact of immunization strategies, public health and social measures on the incidence of varicella among children aged 0-14 years in Quzhou City. Hum Vaccin Immunother. (2026) 22:2639804. doi: 10.1080/21645515.2026.2639804 41773514 PMC12959214

[20] Li X Wang W Kang X Hu P Yu H Lin Y . Vaccine effectiveness and clinical characteristics of breakthrough varicella during outbreaks among students in Qingdao, China. Pediatr Infect Dis J. (2026) 45(6):561–564. doi: 10.1097/INF.0000000000005133 41495930

[21] Liu Y Xu J Zhang H Xu J Zhang Y Wang Q . Impact of the two-dose varicella vaccination recommendation program and surveillance optimization on epidemiological trends in a megacity in China. Hum Vaccin Immunother. (2026) 22:2630521. doi: 10.1080/21645515.2026.2630521 41693349 PMC12915852

[22] Zhang H Liu Y Ye X Luo L He Q . Impact of two-dose varicella vaccination in Guangzhou: an interrupted time-series study. Hum Vaccin Immunother. (2026) 22:2610903. doi: 10.1080/21645515.2025.2610903 41556325 PMC12826739

[23] Li J Huang Y Chen S Song D Chen Y Chang Y . Real-world effectiveness of varicella vaccination in Guangzhou, China, 2017-2022: a matched case-control analysis. Expert Rev Vaccines. (2025) 24:601–11. doi: 10.1080/14760584.2025.2536086 40684305

[24] Bolormaa E Lee YH Choe YJ Choe SA . Varicella vaccine effectiveness and duration of protection: a systematic review and meta-analysis. J Korean Med Sci. (2025) 40:e286. doi: 10.3346/jkms.2025.40.e286 41250651 PMC12624214

[25] Luan G Yao H Yin D Liu J . Trends and age-period-cohort effect on incidence of varicella under age 35 — China, 2005–2021. China CDC Weekly. (2024) 6(18):390–395. doi: 10.46234/ccdcw2024.076 38737482 PMC11082652

[26] Duan C Zhang Y Zhang Q Zhang S Zhong P Gong G . Impact of natural and socio-economic factors on varicella incidence in children in Shanghai, 2013–2022. Front Public Health. (2025) 13:1565717. doi: 10.3389/fpubh.2025.1565717 40496454 PMC12150853

[27] Lin M Yang T Deng P Yang L Xue C . Analysis on the epidemiological characteristics of breakthrough varicella cases and incremental effectiveness of 2-dose varicella vaccine in China. Vaccines (Basel). (2025) 13:160. doi: 10.3390/vaccines13020160 40006707 PMC11860291

[28] Thomas CA Shwe T Bixler D del Rosario M Grytdal S Wang C . Two-dose varicella vaccine effectiveness and rash severity in outbreaks of varicella among public school students. Pediatr Infect Dis J. (2014) 33:1164–8. doi: 10.1097/INF.0000000000000444 24911894 PMC4673889

[29] Watson B Seward J Yang A Witte P Lutz J Chan C . Postexposure effectiveness of varicella vaccine. Pediatrics. (2000) 105:84–8. doi: 10.1542/peds.105.1.84 10617709

[30] Li Y Xu F Liu M Teng S Liang F Wang F . Effectiveness of two-dose vs. single-dose varicella vaccine in children in Shanghai, China: a prospective cohort study. Front Public Health. (2024) 12:1320407. doi: 10.3389/fpubh.2024.1320407 38894987 PMC11183296

[31] Prymula R Bergsaker MR Esposito S Gothefors L Man S Snegova N . Protection against varicella with two doses of combined measles-mumps-rubella-varicella vaccine versus one dose of monovalent varicella vaccine: a multicentre, observer-blind, randomised, controlled trial. Lancet. (2014) 383:1313–24. doi: 10.1016/S0140-6736(12)61461-5 24485548

[32] American Academy of Pediatrics Committee on Infectious Diseases . Prevention of varicella: recommendations for use of varicella vaccines in children, including a recommendation for a routine 2-dose varicella immunization schedule. Pediatrics. (2007) 120:221–31. doi: 10.1542/peds.2007-1089 17606582

